# Perturbations in the blood metabolome up to a decade before prostate cancer diagnosis in 4387 matched case–control sets from the European Prospective Investigation into Cancer and Nutrition

**DOI:** 10.1002/ijc.35208

**Published:** 2024-10-08

**Authors:** Zoe S. Grenville, Urwah Noor, Sabina Rinaldi, Marc J. Gunter, Pietro Ferrari, Claudia Agnoli, Pilar Amiano, Alberto Catalano, María Dolores Chirlaque, Sofia Christakoudi, Marcela Guevara, Matthias Johansson, Rudolf Kaaks, Verena Katzke, Giovanna Masala, Anja Olsen, Keren Papier, Maria‐Jose Sánchez, Matthias B. Schulze, Anne Tjønneland, Tammy Y. N. Tong, Rosario Tumino, Elisabete Weiderpass, Raul Zamora‐Ros, Timothy J. Key, Karl Smith‐Byrne, Julie A. Schmidt, Ruth C. Travis

**Affiliations:** ^1^ Cancer Epidemiology Unit, Oxford Population Health University of Oxford Oxford UK; ^2^ Nutrition and Metabolism Branch International Agency for Research on Cancer, World Health Organization Lyon France; ^3^ Department of Epidemiology and Biostatistics School of Public Health, Imperial College London UK; ^4^ Epidemiology and Prevention Unit, Fondazione IRCCS Istituto Nazionale dei Tumouri Milan Italy; ^5^ CIBER of Epidemiology and Public Health (CIBERESP) Madrid Spain; ^6^ Ministry of Health of the Basque Government, Sub Directorate for Public Health and Addictions of Gipuzkoa San Sebastian Spain; ^7^ BioGipuzkoa (BioDonostia) Health Research Institute, Epidemiology of Chronic and Communicable Diseases Group San Sebastián Spain; ^8^ Centre for Biostatistics, Epidemiology, and Public Health, Department of Clinical and Biological Sciences University of Turin Orbassano Italy; ^9^ Department of Translational Medicine University of Piemonte Orientale Novara Italy; ^10^ Department of Epidemiology Regional Health Council, IMIB‐Arrixaca, Murcia University Murcia Spain; ^11^ Department of Epidemiology and Biostatistics White City Campus, Imperial College London UK; ^12^ Instituto de Salud Pública y Laboral de Navarra Pamplona Spain; ^13^ Centro de Investigación Biomédica en Red de Epidemiología y Salud Pública (CIBERESP) Madrid Spain; ^14^ Navarra Institute for Health Research (IdiSNA) Pamplona Spain; ^15^ Department of Cancer Epidemiology German Cancer research Center (DKFZ) Heidelberg Germany; ^16^ Clinical Epidemiology Unit Institute for Cancer Research, Prevention and Clinical Network (ISPRO) Florence Italy; ^17^ The Danish Cancer Institute Copenhagen Denmark; ^18^ Department of Public Health Aarhus University Aarhus Denmark; ^19^ Escuela Andaluza de Salud Pública (EASP) Granada Spain; ^20^ Instituto de Investigación Biosanitaria ibs.GRANADA Granada Spain; ^21^ Department of Preventive Medicine and Public Health University of Granada Granada Spain; ^22^ Department of Molecular Epidemiology German Institute of Human Nutrition Potsdam‐Rehbruecke Nuthetal Germany; ^23^ Institute of Nutritional Science, University of Potsdam Nuthetal Germany; ^24^ Department of Public Health University of Copenhagen Copenhagen Denmark; ^25^ Hyblean Association for Epidemiology Research, AIRE ONLUS Ragusa Italy; ^26^ International Agency for Research on Cancer, World Health Organization Lyon France; ^27^ Unit of Nutrition and Cancer, Cancer Epidemiology Research Programme, Catalan Institute of Oncology (ICO), Bellvitge Biomedical Research Institute (IDIBELL), L'Hospitalet de Llobregat Barcelona Spain; ^28^ Department of Clinical Epidemiology, Department of Clinical Medicine Aarhus University and Aarhus, University Hospital Aarhus Denmark

**Keywords:** cancer biomarkers, European prospective investigation into cancer and nutrition (EPIC), metabolomics, prospective cohort, prostate cancer

## Abstract

Measuring pre‐diagnostic blood metabolites may help identify novel risk factors for prostate cancer. Using data from 4387 matched case–control pairs from the European Prospective Investigation into Cancer and Nutrition (EPIC) study, we investigated the associations of 148 individual metabolites and three previously defined metabolite patterns with prostate cancer risk. Metabolites were measured by liquid chromatography‐mass spectrometry. Multivariable‐adjusted conditional logistic regression was used to estimate the odds ratio per standard deviation increase in log metabolite concentration and metabolite patterns (OR1SD) for prostate cancer overall, and for advanced, high‐grade, aggressive. We corrected for multiple testing using the Benjamini–Hochberg method. Overall, there were no associations between specific metabolites or metabolite patterns and overall, aggressive, or high‐grade prostate cancer that passed the multiple testing threshold (*padj* <0.05). Six phosphatidylcholines (PCs) were inversely associated with advanced prostate cancer diagnosed at or within 10 years of blood collection. metabolite patterns 1 (64 PCs and three hydroxysphingomyelins) and 2 (two acylcarnitines, glutamate, ornithine, and taurine) were also inversely associated with advanced prostate cancer; when stratified by follow‐up time, these associations were observed for diagnoses at or within 10 years of recruitment (OR_1SD_ 0.80, 95% CI 0.66–0.96 and 0.76, 0.59–0.97, respectively) but were weaker after longer follow‐up (0.95, 0.82–1.10 and 0.85, 0.67–1.06). Pattern 3 (8 lyso PCs) was associated with prostate cancer death (0.82, 0.68–0.98). Our results suggest that the plasma metabolite profile changes in response to the presence of prostate cancer up to a decade before detection of advanced‐stage disease.

## What's New?

Prostate cancer has few known risk factors, but metabolomics may provide some insight in this regard. Here, the authors measured the levels of 148 different individual metabolites, and three metabolite patterns, looking for an association with prostate cancer. They found an association between certain metabolites and a reduced risk of advanced prostate cancer when diagnosed within 10 years of blood collection. One metabolite pattern was associated with prostate cancer death. These results suggest that prostate‐cancer related changes in metabolite profile may occur up to 10 years prior to diagnosis.

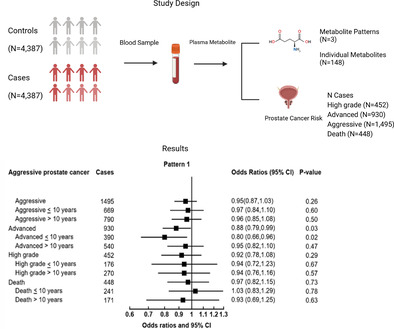

## 1 INTRODUCTION

Prostate cancer is the second leading cause of cancer death in men worldwide.^1^ While few risk factors have been established, metabolomics may be a useful tool for identifying pathways in prostate cancer etiology.^2^, ^3^

Multiple previous studies have investigated circulating metabolites and their associations with more aggressive prostate cancer tumor subtypes; however, these studies have generally lacked statistical power due to limited sample size (<580 advanced cases).^2^, ^4^, ^5^, ^6^, ^7^, ^8^ In the most recent, a case–control study of 3057 matched pairs nested within the European Prospective Investigation into Cancer and Nutrition (EPIC) cohort, identified and assessed three metabolite patterns in relation to prostate cancer risk^2^; a metabolite pattern characterized by 64 diacyl‐ and acyl‐alkyl phosphatidylcholines and three hydroxysphingomyelins, as well as a metabolite pattern of two acylcarntines, glutamate, ornithine, and taurine, were both found to be associated inversely with advanced and aggressive prostate cancer risk. Furthermore, a metabolite pattern of eight lysophosphatidylcholines was observed to be associated inversely with risk of advanced prostate cancer and prostate cancer death.^2^ Additionally, data from other cohorts have reported inverse associations of glycerophospholipids^9^ and acylcarnitine C18:2^4^ with aggressive prostate cancer risk, though data from another cohort reported that six glycerophospholipids were associated positively with aggressive prostate cancer.^4^

We also had an a priori interest in the amino acids based on previous evidence that amino acids, such as branched‐chain and other essential amino acids, and serine, may have an integral role in cancer cell proliferation,^7^, ^10^, ^11^, ^12^ and could be a biomarker in distinguishing more aggressive prostate cancer tumor subtypes.^12^, ^13^

For the current analysis, we expanded our previous dataset considerably from 3057 sets^2^ to 4387 matched sets, with extended median follow‐up from 9.7 to 10.8 years, and with 2477 cases diagnosed more than 10 years after recruitment, compared to 1391 previously. With this larger dataset, we investigated associations of 148 metabolites and three previously determined metabolite patterns^2^ with overall, aggressive, advanced, high grade and death from prostate cancer using the largest prospective sample size to date, stratified at 10 years of follow‐up time.

## 2 METHODS

### 2.1 Study population

The EPIC study is a multi‐center prospective cohort study of >520,000 individuals including 153,400 men, aged mainly between 35 and 70, from 19 centers in eight countries (Denmark, Germany, Greece, Italy, Netherlands, Spain, Sweden, and United Kingdom) who were recruited between 1992 and 2000.^14^ Detailed information on diet and lifestyle was collected at recruitment, and 139,600 men provided a blood sample. Men were eligible for the current study if they had blood stored at the central biobank at the International Agency of Research on Cancer, Lyon, France (IARC; centers in Germany, Italy, Netherlands, Spain, and United Kingdom), or if recruited in Denmark, samples were stored locally only. Further eligibility criteria were that the date of blood collection was known, and no cancer (except non‐melanoma skin cancer) had been diagnosed at the time of blood collection. As a result, data for 4387 cases and 4387 matched controls were available for this study.

### 2.2 Follow up for cases and controls

In Denmark, Italy, the Netherlands, Spain, and the United Kingdom, information on cancer cases, tumor subtypes, and vital status was identified through population cancer registries. In Germany, a combination of methods, such as cancer and pathology registries, health insurance records, and active follow‐up of study subjects were used.^14^

Men diagnosed with prostate cancer (defined as code C61 in the 10th revision of the International Statistical Classification of Diseases and Related Health Problems [ICD‐10]) after blood collection and before the end of follow‐up were categorized as cases.^2^ Each case was matched to one control participant, selected randomly from male cohort participants who were alive and free of cancer (except non‐melanoma skin cancer) at the time the case was diagnosed. An incidence density sampling procedure was used so that a control could become a case at a later date, or be a control for multiple cases. Thus, the OR provides an unbiased estimate of the incidence rate ratio, which would have been obtained from the full cohort.^15^ Matching criteria included study center, length of follow‐up and age (±6 months), time of day (±1 h), and fasting status (<3, 3–6, >6 h) at blood collection.

Prostate cancer subtypes were categorized based on the tumor‐node‐metastasis system and histological grade as follows. Advanced (T3–4 and/or N1–3 and/or M1, or coded as advanced, *n* = 943), high grade (Gleason score 8+ or coded as undifferentiated tumors, *n* = 462), aggressive (advanced, and/or high grade, and/or preoperative PSA >20 ng/mL), and/or death from prostate cancer as the underlying cause of death (*n* = 1495). Overall, 453 men died from prostate cancer as the underlying cause during follow‐up.

### 2.3 Blood collection and laboratory analysis/assay

A standardized protocol was followed for blood collection and processing, and fasting was not required (details published elsewhere).^14^ In brief, for participants from Germany, Italy, the Netherlands, Spain, and the United Kingdom, samples were stored at IARC in plastic straws at −196°C. In Denmark, blood samples were stored in tubes in local repositories, and kept in nitrogen vapor at −150°C. Samples were all assayed at IARC in Lyon, France using the AbsoluteIDQ® p180 Kit (Biocrates Life Sciences AG, Innsbruck, Austria), and following the procedure recommended by Biocrates. To quantify metabolites, liquid chromatography‐mass spectrometry (LC–MS) was applied. All samples were assayed using one LC instrument (Agilent 1290, Santa Clara, CA, USA) coupled with an MS instrument (Triple Quad 4500, AB Sciex, Framingham, MA, USA). Samples from matched case–control sets were assayed in the same analytical batch, along with quality control samples from pooled plasma. Laboratory personnel were blinded to sample category, which includes case, study control, or quality control. A total of 148 metabolites were quantified.

A majority of metabolites (119/148) were measured in all the participants. These were 8 acylcarnitines, 21 amino acids, 5 biogenic amines, 72 phosphatidylcholines (lysophosphatidylcholines [lyso PC, *n* = 8], diacyl phosphatidylcholines [PC aa, *n* = 31] and acyl‐alkyl phosphatidylcholines [PC ae, *n* = 33]), hexose and 12 sphingomyelins (denoted hydroxysphingomyelins [SM (OH), *n* = 5] and sphingomyelins [SM, *n* = 7]). In samples from Denmark, additional data were available for 17 metabolites that had either been excluded in the previous dataset due to not passing quality control thresholds (6 acylcarnitines, 1 amino acid, 2 biogenic amines, 1 lyso PCs, 2 PC aas, and 2 PC aes) or were not previously available (1 lyso PC and 2 SMs). However, we report them here for the Danish data as they passed quality control in the Danish dataset.

### 2.4 Statistical analysis

#### 2.4.1 Individual metabolites

Metabolite measurements were logarithmically transformed for all analyses.

To estimate the risk of prostate cancer per one standard deviation increase in log metabolite concentration, conditional logistic regression was used conditioned on the matching factors. The analysis was further adjusted for exact age at blood collection (continuous), baseline values of body mass index (quartiles; unknown [0.5%]), smoking (never, past, current and unknown [1%]), alcohol intake (<10, 10–19, 20–39 and ≥40 g of alcohol/day; unknown [0.1%]), attained education level (primary, secondary, degree level and unknown [3.3%]) and marital status (married or cohabiting, not married or cohabiting, and unknown [47%]).

The analyses were performed for the full data set (*n* case = 4387) and were also run by tumor subtype (advanced, aggressive, and high grade). To assess the potential for reverse causation in the associations of metabolites with risk of more aggressive prostate cancer stages, analyses for advanced, aggressive, and high‐grade prostate cancer, as well as death from prostate cancer, were further stratified by time to diagnosis (≤10/>10 years); this was done to assess the potential for reverse causality in our analysis of the association of metabolites and metabolite patterns with risk of prostate cancer.

For analyses of individual metabolites, we accounted for multiple testing using the FDR as defined by Benjamini–Hochberg,^16^ with a threshold of 0.05. All tests of statistical significance were two‐sided. *P*‐values after FDR are referred to as *padj* (0.05 was considered as the conventional level for statistical significance).

#### 2.4.2 Metabolite patterns

To compare with our previous EPIC study,^2^ we projected the three treelet components previously identified in 3057 matched sets into the new data from Denmark (1330 matched sets). The treelet components were identified using treelet transform (as described by Gorst‐Rasmussen et al.).^2^, ^17^, ^18^ In summary, treelet transform is a linear dimension‐reduction method that aims to summarize the metabolite concentrations into fewer latent variables, in order to best capture the observed variation in the overall set of metabolites. Full details of the individual metabolites contributing to each metabolite pattern can be found in Supplementary Document 1. Conditional logistic regression using the treelet components as exposure variables was conducted as described for individual metabolites but without correction for multiple testing (*p* <.05).

All analyses were conducted in Stata Statistical Software Package, version 15 (Stata Corporation, College Station, TX).

## 3 RESULTS

Table 1 shows the main characteristics of the participants. On average, participants were 58 years old (SD = 6.5 years) at blood collection. There were no marked differences between cases and controls for selected characteristics.

**TABLE 1:** Characteristics of 4387 prostate cancer patients and 4387 controls from EPIC.

Characteristic	Cases (4387)	Controls (4387)
Age at blood collection, years mean (SD)	57.8 (6.50)	57.7 (6.50)
Height, cm mean (SD)	173.7 (7.15)	173.8 (7.26)
Body mass index, kg/m^2^mean (SD)	26.7 (3.38)	26.8 (3.51)
Smoking, *n* (%)		
Never	1433 (33.0)	1334 (30.7)
Former	1782 (41.1)	1813 (41.7)
Current	1123 (25.9)	1198 (27.6)
Alcohol consumption, *n* (%)		
<10 g/day	1582 (36.2)	1580 (36.0)
10–19 g/day	950 (21.7)	958 (21.8)
20–40 g/day	961 (22.0)	991 (22.6)
≥40 g/day	883 (20.2)	857 (19.5)
Physical activity, *n* (%)		
Inactive	852 (19.7)	887 (20.5)
Moderately inactive	1350 (31.3)	1362 (31.5)
Moderately active	1028 (23.8)	1030 (23.8)
Active	1090 (25.2)	1045 (24.2)
Marital status, *n* (%)		
Married or cohabiting	2051 (88.5)	2063 (88.9)
Not married or cohabiting	267 (11.5)	257 (11.1)
Educational attainment, *n* (%)		
Primary or equivalent	1637 (38.6)	1615 (38.0)
Secondary	1489 (35.2)	1529 (36.0)
Degree	1110 (26.2)	1107 (26.0)
Cases only		
Year of diagnosis, median(range)	2006 (1994–2012)	
Time to diagnosis, *n* (%)		
<5 years	493 (11.5)	
5–10 years	1316 (30.7)	
10–15 years	1904 (44.4)	
≥15 years	573 (13.4)	
Data source, *n* (%)		
Schmidt et al. (2020)	3057 (69.7%)	3057 (69.7%)
New Danish data	1330 (30.3%)	1330 (30.3%)

### 3.1 Individual metabolites

There were no associations of the individual metabolites, including the amino acids, with overall, aggressive, high‐grade prostate cancer, prostate cancer death, or advanced prostate cancer before stratification by follow‐up time, after adjustment for multiple testing.

**FIGURE 1:**
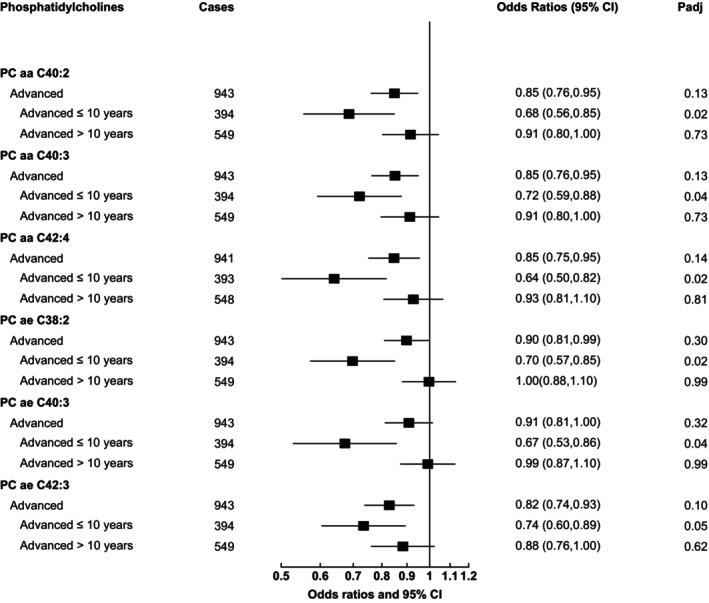
Odds ratios of advanced prostate cancer by concentration of six phosphatidylcholines. Odds ratios of advanced prostate cancer associated with a one standard deviation increase in concentration of six PCs. Stage and grade of prostate cancer were categorized using the tumor‐node‐metastasis (TNM) system and Gleason score, respectively; advanced (T_3–4_ and/or N_1–3_ and/or M_1_, or coded as advanced. CI, confidence interval; OR_1SD_, odds ratio for a one standard deviation increase in metabolite concentration.

For men with advanced prostate cancer diagnosed at or within 10 years of blood collection, the association with PC aas C42:4 (OR_1SD_ = 0.64, 95% CI 0.50–0.82, *p*‐value = .0003, *padj* = 0.02), C40:2 (OR_1SD_ = 0.68, 95% CI 0.56–0.85, *p*‐value = .0005, *padj* = 0.02), C40:3 (OR_1SD_ = 0.72, 95% CI 0.59–0.88, *p*‐value = .001, *padj* = 0.04), and PC aes C38:2 (OR_1SD_ = 0.70, 95% CI 0.57–0.85, *p*‐value = .0003, *padj* = 0.02), C40:3 (OR_1SD_ = 0.67, 95% CI 0.53–0.86, *p*‐value = .001, *padj* = 0.04), and C42:3 (OR_1SD_ = 0.74, 95% CI 0.60–0.89, *p*‐value = .002, *padj* = 0.05) passed correction for multiple testing(FDR <0.05) (see Figure 1).

Of the amino acids, eight (serine, threonine, aspartic acid, histidine, glutamine, proline, arginine, and asparagine) were conventionally significantly (*p*‐value <.05) associated with at least one prostate cancer outcome (Supplementary Document 2). The strongest association was observed for serine, which associated positively with aggressive prostate cancer risk (OR_1SD_ = 1.10, 95% CI 1.01—1.19, *p*‐value = .02, *padj* = 0.49), including for diagnoses more than 10 years after blood collection (OR_1SD_ = 1.20, 95% CI 1.07–1.35, *p*‐value = .002, *padj* = 0.31).

### 3.2 Metabolite patterns and prostate cancer risk

**FIGURE 2:**
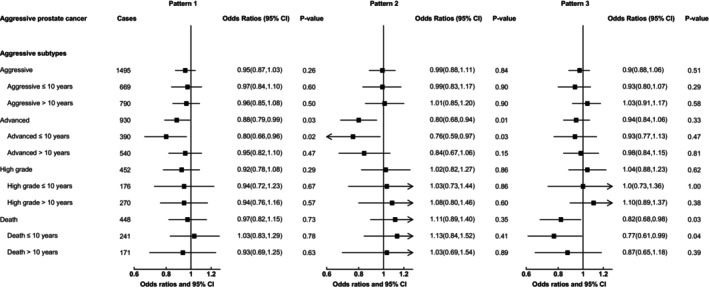
Odds ratio of prostate cancer subgroups associated with a one standard deviation increase in metabolite pattern scores. Odds ratio of prostate cancer subtypes associated with a one standard deviation increase in metabolite pattern scores. Stage and grade of prostate cancer were categorized using the tumor‐node‐metastasis (TNM) system and Gleason score, respectively; advanced (T_3–4_ and/or N_1–3_ and/or M_1_, or coded as advanced), high grade (Gleason 8+ or coded as undifferentiated tumors), and aggressive (advanced, high grade, and death combined). Death from prostate cancer during follow‐up was defined as prostate cancer listed as the underlying cause of death on the death certificate. CI, confidence interval; OR_1SD_, odds ratio for a one standard deviation increase in metabolite pattern score.

Figure 2 and Supplementary Document 3 show associations of metabolite patterns and risk of prostate cancer. There were no associations found for prostate cancer overall, nor with aggressive or high‐grade prostate cancer.

Metabolite pattern 1 (characterized by higher concentrations of diacyl and acyl‐alkyl phosphatidylcholines and three hydroxysphingomyelins) was associated inversely with advanced‐stage prostate cancer risk (odds ratio for an SD increase in treelet component score OR_1SD_ = 0.88, 95% CI 0.79–0.99, *p*‐value = .03). When stratifying by follow‐up time, the association only remained for men diagnosed at or within 10 years of blood collection (OR_1SD_ = 0.80, 95% CI 0.66–0.96, *p*‐value = .02), while for men diagnosed later, the odds ratio was 0.95 (95% CI 0.82–1.10, *p*‐value = .47).

Pattern 2, which comprised higher acylcarnitines C18:1 and C18:2, glutamate, ornithine, and taurine, was also associated inversely with advanced‐stage prostate cancer risk (OR_1SD_ = 0.80, 95% CI 0.68–0.94, *p*‐value = .008). Similar to Pattern 1, the association was stronger in men with 10 or fewer years of follow‐up (OR_1SD_ = 0.76, 95% CI 0.59–0.97, *p*‐value = .03), than for cases diagnosed later (OR_1SD_ = 0.84, 95% CI 0.67–1.06, *p*‐value = .15).

Men who scored higher on Pattern 3, which is characterized by eight lysophosphatidylcholines, had a lower risk of prostate cancer death (OR_1SD_ = 0.82, 95% CI 0.68–0.98, *p*‐value = .03). In those diagnosed at or within 10 years, the odds ratio was 0.77 (0.61, 0.99, *p*‐value = .04), and in those diagnosed after longer follow‐up, the odds ratio was 0.87 (0.65, 1.18, *p*‐value = .39).

## 4 DISCUSSION

In this large prospective case–control study of plasma metabolites and prostate cancer risk, there were associations observed between 3 PC aas and 3 PC aes and lower risk of advanced prostate cancer, in men diagnosed at or within 10 years of blood collection. For the amino acids, serine was associated with an increased risk of aggressive prostate cancer after 10 years of follow‐up, though this association did not remain after correction for multiple testing. Finally, using three metabolite patterns that were previously identified,^2^ we observed a lower risk of advanced‐stage prostate cancer in men with metabolite profiles characterized by higher concentrations of PCs and SM(OH)s (Pattern 1) and acylcarnitines C18:1 and C18:2, glutamate, ornithine and taurine (Pattern 2), diagnosed at or within 10 years of blood collection. Furthermore, higher levels of Pattern 3 (8 lyso PCs) were associated with lower risk of death from prostate cancer.

Previously in EPIC, we investigated metabolite patterns and prostate cancer, using a subset of the current data (~70%).^2^ In this earlier analysis, we reported that Patterns 1 and 2 were inversely associated with advanced prostate cancer overall, however, in our current extended analysis this association does not persist in men diagnosed more than 10 years after blood collection. PC aas C40:2, C40:3, and C42:4, which were associated with men diagnosed with advanced‐stage disease at or within 10 years of blood collection in our individual metabolites analysis, were among several PC aas that were associated with lower risk of advanced prostate cancer previously in EPIC.^2^ However, in this previous study associations were similar in cases by duration of follow‐up. Similarly, higher levels of several PC aes were associated with lower risk of advanced prostate cancer in the previous study, among them PC aes C38:2 and C42:3, which were associated with advanced disease in men with up to 10 years of follow‐up in our current individual metabolites analyses.^2^

Finally, in line with our current findings, our previous study found Pattern 3 to be associated with lower risk of death from prostate cancer.^2^ In our current analyses by follow‐up time, however, Pattern 3 was associated with death only in men diagnosed within 10 years of blood collection, though there were a relatively small number of deaths limiting the power of the stratified analyses.

The weaker association in the subgroups with longer follow‐up, despite the now larger sample size and greater power to detect an association, suggests that the associations observed may be due to reverse causation, with the presence of pre‐clinical cancer altering metabolite levels.^14^

Comparing these results to other prospective cohort studies is complex, due to differing analytical platforms and different classifications of aggressive prostate cancer tumor subtypes. Though there are no other studies that applied dimension reduction methods to the full set of metabolites available in this EPIC study, a principal component analysis (PCA) conducted in the Alpha‐Tocopherol, Beta‐Carotene Cancer Prevention Study (ATBC) reported that the first principal components of metabolites in pathways of uracil‐containing pyrimidine, dipeptide, glycine/serine/threonine, gamma‐glutamyl amino acid, aminosugar, polyunsaturated fatty acid (n3 and n6), and endocannabinoid metabolism were associated positively with risk of overall lethal prostate cancer.^5^ In a gene set analysis, the ATBC study reported strong inverse associations between lipid and energy metabolite chemical classes and aggressive cancer.^7^ Meanwhile, the Prostate, Lung, Colorectal, and Ovarian Cancer Screening Trial did not find any of their 10 calculated principal components to have an association with aggressive prostate cancer.^8^ Regarding individual metabolite analyses, in the ATBC study higher glycerophospholipid concentrations were associated with lower risk of tumor stage T3 prostate cancer.^5^ In contrast, a nested case–control in the Northern Sweden Health and Disease Study (NSHDS) found that higher concentrations of three phosphatidylcholines (PC aes C38:3, C38:4, and C40:2) were associated with increased aggressive prostate cancer risk, which included tumor stage T3.^4^ There have been few studies on the metabolites loading on Pattern 2 and their associations with risk of advanced prostate cancer.

The current analyses found that men with higher scores of Pattern 3 (8 Lyso PCs) had a lower risk of death from prostate cancer. In line with this, in an ATBC study, higher levels of lysolipid 1‐linoleoyl‐glycerophosphatidylcholine (18:2) (which is comparable to lysoPC C18:2 included in Pattern 3) were also associated with reduced risk of prostate cancer death.^5^ In contrast, in the NSHDS, three Lyso PCs (C17:0, C20:3, and C20:4) were associated positively with aggressive prostate cancer risk.^4^

Our results by time to diagnosis suggest that the associations with advanced disease are likely due to the influence of prostate cancer on the circulating metabolome, rather than the circulating metabolome affecting risk of prostate cancer development. This is in line with findings of marked changes in the metabolite profile of men with prostate cancer (as reviewed by Kelly et al. (2016) and Lima et al. (2016)), including in circulating levels of glycerophospholipids and amino acids.^19^, ^20^

Many of the measured metabolites have been implicated in post‐diagnostic prostate cancer studies. For example, in a review of metabolomics biomarkers of prostate cancer, the author noted that there was evidence of glutamate and taurine in distinguishing malignant from benign prostatic tissue in a magnetic resonance spectroscopy (NMR) study^19^; a more recent NMR study also found increased glutamate levels in tumors with progressively higher Gleason scores.^21^ Men diagnosed with prostate cancer have also been found to have altered levels of acylcarnitines, lyso PCs, and PCs.^20^, ^22^

Following up on an a priori interest in the amino acids, none of the amino acids were significantly associated with prostate cancer subtypes after correction for multiple testing. However, serine was positively associated at conventional significance with aggressive disease in men with more than 10 years of follow‐up; chance cannot be excluded as a possible explanation, and replication and a larger sample size would be needed to further investigate this. Several laboratory studies have suggested that serine may have an integral role in cancer cell proliferation,^12^ and maybe a biomarker in distinguishing more aggressive prostate cancer tumor subtypes.^13^ Serine is strongly interconnected with glycine metabolism, which has been implicated in cancer cell growth and as a plausible predictor of metastatic prostate cancer.^23^, ^24^, ^25^ Furthermore, a case–control analysis that pooled five different cancers (prostate included) observed that plasma‐free amino acids, including serine, were present in higher concentrations in those who had cancer.^26^

### 4.1 Strengths and limitations

Due to the large cohort size, this analysis provided greater statistical power than prior prospective studies, including our previous analysis, especially for analyses by prostate cancer subtype and time to diagnosis. For instance, we were particularly interested in advanced prostate cancer; for men with advanced disease, there were 350 additional cases in the current study, including 62 new cases for men diagnosed within 10 years of blood collection, and 288 new cases for men with advanced disease after 10 years of blood collection.^2^ Furthermore, using both individual metabolites and metabolite patterns as exposure variables allowed for the identification of specific metabolite risk associations, as well as investigation of the associations with groups of correlated metabolites that might implicate potential metabolic pathways.

Limitations include different sample handling procedures between study centers, as well as the use of non‐fasting blood samples, both of which may impact measured metabolite levels.^27^, ^28^ In order to mitigate these effects, cases and controls were matched on study center and fasting status.^29^ Furthermore, only one blood sample was available per participant, which may lead to attenuation of risk estimates if a single measurement does not represent long‐term exposure.^30^, ^31^

Our work on an extended dataset emphasizes the importance of a large sample size for stable risk estimates. These results may have implications for other nested case–control studies of metabolites and cancer types.

## 5 CONCLUSION

These findings suggest that the results of our previous study between circulating metabolites and prostate cancer risk may be explained by the effects of pre‐clinical disease. We identified several associations between metabolites and risk of advanced prostate cancer among men diagnosed in the first 10 years after blood collection. These results may indicate that the metabolite profile of blood starts to change in response to prostate cancer up to a decade before the cancer is detected at an advanced stage.

## AUTHOR CONTRIBUTIONS

**Zoe S. Grenville:** Conceptualization; formal analysis; methodology; writing – original draft; writing – review and editing. **Urwah Noor:** Methodology; writing – review and editing. **Sabina Rinaldi:** Resources; writing – review and editing. **Marc J. Gunter:** Resources; writing – review and editing. **Pietro Ferrari:** Resources; writing – review and editing. **Claudia Agnoli:** Resources; writing – review and editing. **Pilar Amiano:** Resources; writing – review and editing. **Alberto Catalano:** Resources; writing – review and editing. **María Dolores Chirlaque:** Resources; writing – review and editing. **Sofia Christakoudi:** Resources; writing – review and editing. **Marcela Guevara:** Resources; writing – review and editing. **Matthias Johansson:** Resources; writing – review and editing. **Rudolf Kaaks:** Resources; writing – review and editing. **Verena Katzke:** Resources; writing – review and editing. **Giovanna Masala:** Resources; writing – review and editing. **Anja Olsen:** Resources; writing – review and editing. **Keren Papier:** Writing – review and editing. **Maria‐Jose Sánchez:** Resources; writing – review and editing. **Matthias B. Schulze:** Resources; writing – review and editing. **Anne Tjønneland:** Resources; writing – review and editing. **Tammy Y. N. Tong:** Writing – review and editing. **Rosario Tumino:** Resources; writing – review and editing. **Elisabete Weiderpass:** Resources; writing – review and editing. **Raul Zamora‐Ros:** Resources; writing – review and editing. **Timothy J. Key:** Conceptualization; funding acquisition; methodology; resources; supervision; writing – original draft; writing – review and editing. **Karl Smith‐Byrne:** Conceptualization; methodology; supervision; writing – original draft; writing – review and editing. **Julie A. Schmidt:** Conceptualization; methodology; resources; supervision; writing – original draft; writing – review and editing. **Ruth C. Travis:** Conceptualization; funding acquisition; methodology; resources; supervision; writing – original draft; writing – review and editing.

## FUNDING INFORMATION

This work was supported by Cancer Research UK [C8221/A30904 and C8221/A29017]. The coordination of EPIC‐Europe is financially supported by International Agency for Research on Cancer (IARC) and also by the Department of Epidemiology and Biostatistics, School of Public Health, Imperial College London which has additional infrastructure support provided by the NIHR Imperial Biomedical Research Centre (BRC). The national cohorts are supported by: Danish Cancer Society (Denmark); Ligue Nationale Contre le Cancer, Institut Gustave Roussy, Mutuelle Générale de l'Education Nationale (MGEN), Institut National de la Santé et de la Recherche Médicale (INSERM), French National Research Agency (ANR, reference ANR‐10‐COHO‐0006), French Ministry for Higher Education (subsidy 2102918823, 2103236497, and 2103586016) (France); German Cancer Aid, German Cancer Research Center (DKFZ), German Institute of Human Nutrition Potsdam‐Rehbruecke (DIfE), Federal Ministry of Education and Research (BMBF) (Germany); Associazione Italiana per la Ricerca sul Cancro‐AIRC‐Italy, Italian Ministry of Health, Italian Ministry of University and Research (MUR), Compagnia di San Paolo (Italy); “Europe against Cancer” Programme of the European Commission (DG SANCO); Dutch Ministry of Public Health, Welfare and Sports (VWS), the Netherlands Organisation for Health Research and Development (ZonMW), World Cancer Research Fund (WCRF), (The Netherlands); UiT The Arctic University of Norway; Health Research Fund (FIS)—Instituto de Salud Carlos III (ISCIII), Regional Governments of Andalucía, Asturias, Basque Country, Murcia and Navarra, and the Catalan Institute of Oncology—ICO (Spain); Swedish Cancer Society, Swedish Research Council and County Councils of Skåne and Västerbotten (Sweden); Cancer Research UK (C864/A14136 to EPIC‐Norfolk; C8221/A29017 to EPIC‐Oxford), Medical Research Council (MR/N003284/1, MC‐UU_12015/1 and MC_UU_00006/1 to EPIC‐Norfolk; MR/Y013662/1 to EPIC‐ Oxford) (United Kingdom). The funders had no role in the design of the study; in the collection, analyses, or interpretation of data; in the writing of the manuscript; or in the decision to publish the results.

## CONFLICT OF INTEREST STATEMENT

The authors declare no conflict of interest.

## ETHICS STATEMENT

All participants in the EPIC study provided written informed consent, and the study was approved by the IARC Ethics Committee (ref IEC 14‐02) and the ethical review boards of all local institutions where participants had been recruited.

## Supporting information


**Data S1:** Supporting Information

**Data S2:** Supporting Information

**Data S3:** Supporting Information

## Data Availability

EPIC data are available for investigators who seek to answer important questions on health and disease in the context of research projects that are consistent with the legal and ethical standard practices of IARC/WHO and the EPIC Centers. The primary responsibility for accessing the data belongs to the EPIC centers that provided them. For information on how to submit an application for gaining access to EPIC data and/or biospecimens, please follow the instructions at http://epic.iarc.fr/access/index.php. Further information is available from the corresponding author upon request.
