# Supplementary material for: Perturbations in the blood metabolome up to a decade before prostate cancer diagnosis in 4387 matched case–control sets from the European Prospective Investigation into Cancer and Nutrition
Source: Int J Cancer. 2024 Oct 8;156(5):943–52. doi: 10.1002/ijc.35208 (PMC11701393; doi:10.1002/ijc.35208)
Supplement: Supplementary file 1 — Data S1: Supporting Information [file IJC-156-943-s002.pdf]

Perturbations in the blood metabolome up to a decade before prostate cancer diagnosis in 4,387 matched case-control sets from the European Prospective Investigation into Cancer and Nutrition

SUPPORTING INFORMATION

Zoe S Grenville, Urwah Noor, Sabina Rinaldi , Marc J Gunter, Pietro Ferrari, Claudia Agnoli, Pilar Amiano, Alberto Catalano, María Dolores Chirlaque, Sofia Christakoudi, Marcela Guevara, Matthias Johansson, Rudolf Kaaks, Verena Katzke, Giovanna Masala, Anja Olsen, Keren Papier, Maria-Jose Sánchez, Matthias B Schulze, Anne Tjønneland, Tammy YN Tong, Rosario Tumino, Elisabete Weiderpass, Raul Zamora-Ros, Timothy J Key, Karl Smith-Byrne, Julie A Schmidt, and Ruth C Travis

Contents:

Supplementary Document 1. Individual Metabolites Loading on Each Metabolite Pattern 2
Supplementary Document 2……………………………………………….Please see excel file
Supplementary Document 3……………………………………………….Please see excel file

Supplementary Document 1. Individual Metabolites Loading on Each Metabolite Pattern

Metabolite Pattern	Contributing Metabolites
	**Diacyl-alkyl-phosphatidylcholines:**
1	PC aa C28:1
	PC aa C30:0
	PC aa C32:0
	PC aa C32:1
	PC aa C32:3
	PC aa C34:1
	PC aa C34:2
	PC aa C34:3
	PC aa C34:4
	PC aa C36:0
	PC aa C36:1
	PC aa C36:2
	PC aa C36:3
	PC aa C36:4
	PC aa C36:5
	PC aa C36:6
	PC aa C38:0
	PC aa C38:3
	PC aa C38:4
	PC aa C38:5
	PC aa C38:6
	PC aa C40:2
	PC aa C40:3
	PC aa C40:4
	PC aa C40:5
	PC aa C40:6
	PC aa C42:0
	PC aa C42:1
	PC aa C42:2
	PC aa C42:4
	PC aa C42:5
	**Acyl-alkyl-phosphatidylcholines:**
	PC ae C30:0
	PC ae C30:2
	PC ae C32:1
	PC ae C32:2
	PC ae C34:0
	PC ae C34:1
	PC ae C34:2
	PC ae C34:3
	PC ae C36:0
	PC ae C36:1
	PC ae C36:2
	PC ae C36:3

Metabolite Pattern	Contributing Metabolites
	PC ae C36:4<br>PC ae C36:5<br>PC ae C38:2<br>PC ae C38:3<br>PC ae C38:4<br>PC ae C38:5<br>PC ae C38:6<br>PC ae C40:1<br>PC ae C40:2<br>PC ae C40:3<br>PC ae C40:4<br>PC ae C40:5<br>PC ae C40:6<br>PC ae C42:1<br>PC ae C42:2<br>PC ae C42:3<br>PC ae C42:4<br>PC ae C42:5<br>PC ae C44:4<br>PC ae C44:5<br>PC ae C44:6<br>**Hydroxysphingomyelins:**<br>SM (OH) C14:1<br>SM (OH) C16:1<br>SM (OH) C22:2
2	**Acylcarnitines:**<br>C18:1<br>C18:2<br><br>**Amino acids:**<br>Glutamate<br>Ornithine<br><br>**Biogenic Amine:**<br>Taurine
3	**Lysophosphatidylcholines:**<br>Lyso PC a C16:0<br>Lyso PC a C16:1<br>Lyso PC a C17:0<br>Lyso PC a C18:0<br>Lyso PC a C18:1<br>Lyso PC a C18:2<br>Lyso PC a C20:3<br>Lyso PC a C20:4
